# The Morphological Diversity of Plant Organs: Manipulating the Organization of Microtubules May Do the Trick

**DOI:** 10.3389/fcell.2021.649626

**Published:** 2021-03-26

**Authors:** Zhiru Bao, Zhijing Xu, Jingze Zang, Katharina Bürstenbinder, Pengwei Wang

**Affiliations:** ^1^Key Laboratory of Horticultural Plant Biology (MOE), College of Horticulture and Forestry Sciences, Huazhong Agricultural University, Wuhan, China; ^2^Interdisciplinary Sciences Research Institute, Huazhong Agricultural University, Wuhan, China; ^3^National R&D Centre for Citrus Preservation, Huazhong Agricultural University, Wuhan, China; ^4^Department of Molecular Signal Processing, Leibniz Institute of Plant Biochemistry, Halle (Saale), Germany

**Keywords:** cytoskeleton, microtubules, cell morphogenesis, organ shape, IQD, ROP

## Plant Microtubules and Microtubule-Associated Proteins

**Figure 1:**
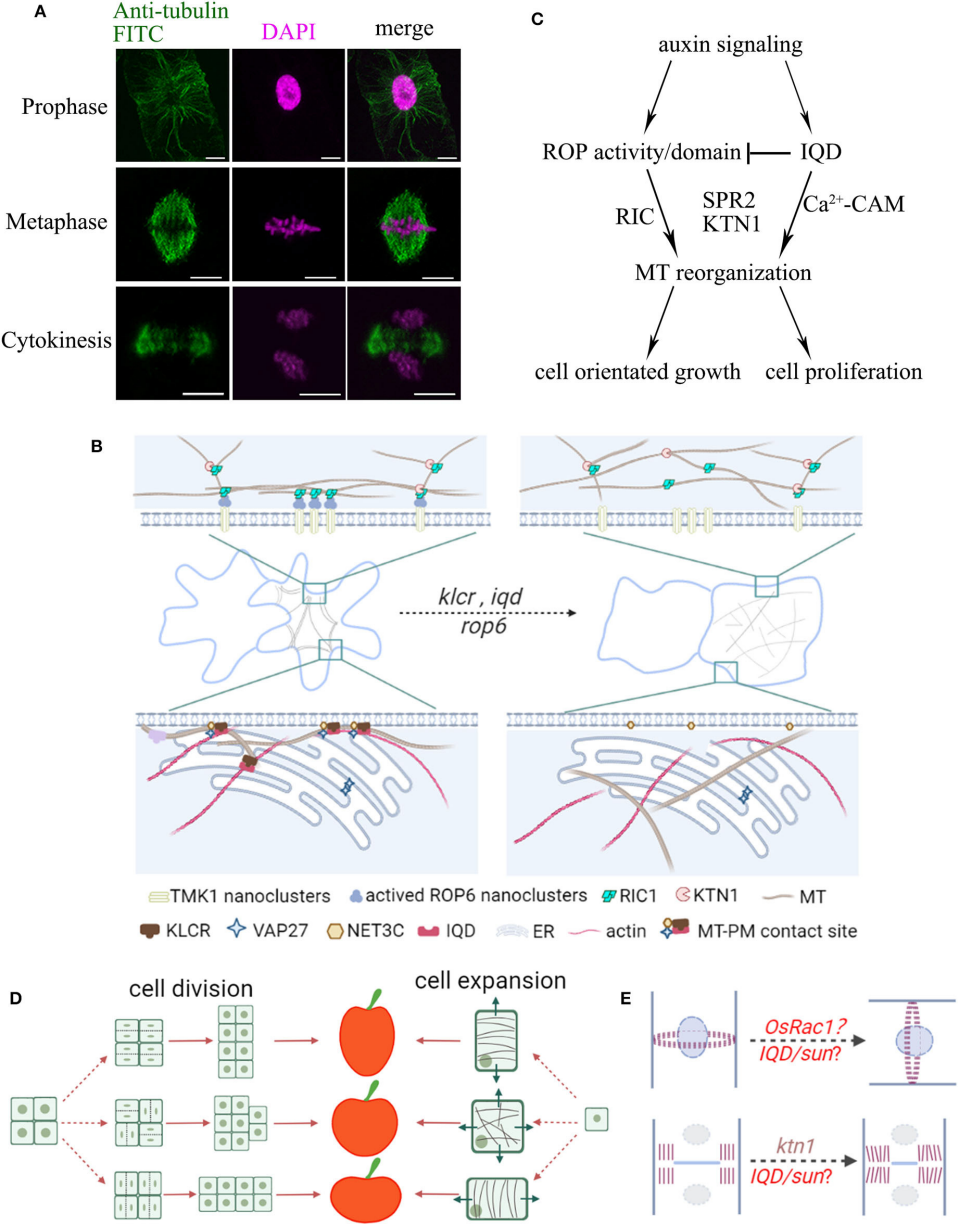
Schematic summary of the organization of plant organ shape regulated by microtubules. **(A)** Localization of microtubules during cell division. BY-2 cells were immuno-labeled with anti-tubulin (microtubules, green) and DAPI (magenta, DNA). Microtubules rearrange throughout prophase, metaphase, and telophase to form the preprophase band (PPB), spindle apparatus, and phragmoplast, respectively. Bars, 10 μm. **(B)** Leaf pavement cells display a typical jigsaw puzzle-shape appearance. During lobe formation, cortical microtubules are enriched at convex neck regions. MT-PM contact sites may take part in reorganization of microtubule arrays through IQDs-KLCR and ROP6-RIC1 proteins, whose mutants show more circular pavement cells and disorganized cytoskeletal networks. **(C)** Model of ROP and IQD pathways and their (proposed) role in regulating cell growth and division. Auxin signals activate ROPs, and recruit RIC1, KTN1 or other MAPs to promote microtubule ordering. On the other hand, auxin signals also activate IQDs, which regulate the distribution of ROPs on the PM and the organization of microtubules, and direct the orientation of cell growth and division. **(D)** Fruit shapes are controlled by the direction of cell division and cell expansion. During anisotropic growth, the orientation of cortical microtubules is perpendicular to the direction of cell expansion. For example, in elongated fruits, cell expansion is likely oriented longitudinally and microtubules are aligned transversely. **(E)** The position of the PPB marks the site of cell division. The positional information is maintained by a polarized membrane domain, termed CDZ. The new cell wall is inserted at the cell center by the phragmoplast, which guides the growing cell plate toward the CDZ. The direction of cell division will greatly influence organ shape. Altered expression of ROPs may affect division plane positioning. Mutants in *KTN1* display delayed phragmoplast growth. Division plane determination may further be controlled by other shape regulators such as IQDs or other MAPs.

The plant cytoskeleton is a highly dynamic filamentous system and plays important roles in various intracellular processes including cell division, intracellular trafficking, immune responses, and stress tolerance (Li and Staiger, 2018; Livanos and Müller, 2019). Although both actin and microtubules are essential in the determination of cell morphology and organ shape, in this article we will mainly focus on microtubules, which are regulated by various microtubule-associated proteins (MAPs). Aberrant expression of MAPs affects microtubule organization and consequently cell function and morphogenesis (Ruan et al., 2018). Microtubules are essential in cell wall formation by guiding the directional movement of cellulose synthase complexes in the plasma membrane (PM) (Paredez et al., 2006). Perturbation of microtubule functions often leads to changes in cell wall composition and cell stiffness, ultimately affecting cell expansion and plant architecture. Loss- or gain-of-function in several MAPs and MT-related proteins, e.g., IQ67-Domain proteins (IQD) and Rho of plant GTPases (ROPs), leads to anisotropic or helical growth phenotypes in petals, cotyledons and hypocotyls, which are attributed to the alteration of microtubule organization (Yang et al., 2019; Zang et al., 2021). In dividing plant cells, microtubules form unique structures such as the preprophase band, the acentrosomal mitotic spindle, and the phragmoplast, essential for cell plate directional expansion (Figure 1A).

Although the structure and function of microtubules is similar across kingdoms, plants have evolved specific regulatory mechanisms to coordinate cytoskeletal functions in response to incoming signals. Within these diverse signaling pathways, calcium ions (Ca^2+^) serve as universal second messengers and several studies implicate roles of Ca^2+^ in regulation of the cytoskeleton (Hepler, 2016). Ca^2+^ signals may be integrated at microtubules via differential binding of calmodulin (CaM)-Ca^2+^ sensors to several MAPs (Kölling et al., 2019). One class of CaM targets are IQDs, which are plant-specific microtubule binding proteins that may recruit CaM to microtubules and other specific subcellular compartments in response to Ca^2+^ signals to coordinate plant development and cell shape formation (Bürstenbinder et al., 2013, 2017; Zang et al., 2021).

## The Regulation of Cortical Microtubules in Leaf Pavement Cell Shape

Leaf pavement cells emerged as a popular model system to study the formation of complex cell shapes. In several species, including *Arabidopsis*, pavement cells display an interlocking jigsaw puzzle-shape appearance, whose development relies on polarity establishment and is influenced by the cytoskeleton, hormonal signals, and mechanical stress (Bidhendi et al., 2019; Pan et al., 2020). The removal of actin filaments or actin regulating proteins (e.g., ARP2/3; SCAR/WAVE) leads to smaller lobes (Cifrová et al., 2020) suggesting that cortical actin filaments that localize to the growing lobe tips may regulate lobe outgrowth. In pavement cells, cortical microtubules are persistently enriched in periclinal walls at the convex side of lobes and mutants defective in MAPs often display defects in lobe formation and/or growth (Armour et al., 2015; Wong et al., 2019). Microtubules thus may generate a patch of anisotropic strain or guide a local thickening of the cell wall to restrict growth and promote lobe formation (Figure 1B) (Altartouri et al., 2019; Wong et al., 2019).

A recent study proposed the role of auxin gradient in regulating the pavement cell pattern through transmembrane kinase 1 (TMK1)-dependent ROP6 nanoclustering at the PM (Grones et al., 2020). The activated ROP6 nanoclusters recruit RIC1 (ROP-interactive CRIB motif-containing protein 1) effector proteins, which localize at cortical microtubules and interact with Katanin1 (KTN1) to promote microtubule ordering and lead to the formation of indentations (Figures 1B,C) (Ren et al., 2017; Pan et al., 2020). Interestingly, IQD13, a protein that interacts with cortical microtubules and the PM, regulates the distribution of active ROP domains on the PM in differentiating metaxylem cells (Sugiyama et al., 2017). Therefore, it is likely that IQD proteins may interact with other MAPs and regulate ROP activity in non-xylem cells in a similar way, such as IQD5 that is also enriched at indentation sites (Figure 1C) (Liang et al., 2018; Mitra et al., 2019; Li et al., 2020).

In contrast, other work supports a turgor-driven mechanical model for the regulation of interlocking patterns. The localized mechano-stress asymmetry could trigger the local MT rearrangement and subsequent local cellulose deposition coupled with de-esterified pectin, collectively contributing to local cell wall reinforcement, outgrowth restriction, and the formation of indentation regions (Majda et al., 2017; Bidhendi et al., 2019). Therefore, the interplay among plant hormones, microtubule regulators and mechano-sensing are complicated but exciting stories for future studies.

In addition, it is known that the structure of microtubules or actin can be influenced by each other. Cells with dysfunctional actin networks also exhibit alternations in microtubule organization (Cifrová et al., 2020). Our recent study has revealed a plant specific actin-microtubule bridging complex, consisting of Networked protein 3C (NET3C), Kinesin Light Chain-Related/Cellulose Microtubule Uncoupling1 (KLCR1/CMU1) and IQDs. These proteins localize to membrane interfaces, and cross link the ER network, PM, actin cytoskeleton, and microtubules (Zang et al., 2021). Their respective mutants all exhibit defects in pavement cell morphogenesis, implicating a function of membrane contact sites and the cytoskeleton-membrane interface in microtubule regulation (Figure 1B). Therefore, as the ER, PM, and cytoskeleton are closely associated, affecting membrane lipid or protein composition may also affect cytoskeleton organization, producing a net effect on cell morphologies.

## Microtubule Regulating Proteins Are Common Genetic Traits for Morphological Diversity of Crop Plants

Likewise, the shape determination of other plant organs is likely conserved, and fruit (or seed) shape establishment is a good example. Fruits exhibit a great diversity, from simple spherical and cylindrical structures to more complex shapes. Their morphogenesis is tightly controlled by cell expansion and cell division (Figure 1D). In agreement with this hypothesis, recent discoveries indicate that genes encoding microtubule related proteins are regularly identified to affect fruit shape from independent genetic mapping studies (Wu et al., 2018; Yang et al., 2020). A prominent example are IQD proteins, which emerged as key regulators of organ morphogenesis during domestication. In tomato, watermelon, cucumber, and rice, the expression level of IQDs is positively associated with elongated fruit/seed shape (Wu et al., 2011; Pan et al., 2017; Dou et al., 2018; Yang et al., 2020).

In rice, the expression level of OsIQD14 or OsIQD26 proteins, which act in auxin and brassinosteroid signaling pathways respectively, are directly related to the weight and length of grains, possibly by regulating cell proliferation in spikelet hulls through a microtubule dependent pathway (Figure 1C, Duan et al., 2017; Yang et al., 2020). Similarly, the tomato *sun* mutant, which exhibits higher expression of *SlIQD12*, has longer fruits than the wild type, producing excessive longitudinal cell divisions and decreased cell division in the transverse direction. This suggests that changes in the cell division patterns and rearranging directional cell expansion contribute significantly to fruit elongation (Wu et al., 2011). A recent study found that OsRac1, a member of ROP-GTPases, controls rice grain size and yield by promoting cell division (Zhang et al., 2019). In maize, *rop* mutants show asymmetric cell division in guard cells, due to the disruption of cortical division zone positioning (Humphries et al., 2011). Intriguingly, several IQD proteins identified to date localize to interphase microtubules and mitotic microtubules, including the preprophase band, mitotic spindle, or phragmoplast (Bürstenbinder et al., 2017; Liang et al., 2018). Therefore, ROP and IQD proteins may be involved in determination of cell division patterns in crop fruits and other plant organs (Figure 1E).

IQD proteins share hallmarks of scaffold proteins that interact with multiple MAPs, likely providing a platform for macromolecular complex assemblies that regulate the microtubule structure (Bürstenbinder et al., 2013, 2017). A recent study showed that the *Arabidopsis* MAP, Spiral2 (SPR2) physically interacts with IQD18, and this interaction is reduced in the presence of Ca^2+^ (Wendrich et al., 2018). KTN1 severs microtubules at crossovers and promotes microtubule bundle formation. Free SPR2 may bind to microtubule minus ends and promote KTN1-dependent severing, resulting in increased microtubule dynamics (Nakamura et al., 2018; Wendrich et al., 2018). Similarly, IQDs may control cell division orientation by coordinating the severing activity of KTN1 during cytokinesis (Komis et al., 2017; Li et al., 2020). Moreover, phenotypic studies of *ktn1* mutants showed defective organization of mitotic microtubule arrays, delayed cytokinetic progression, and unstable PPB organization, all of which lead to excessive longitudinal cell divisions at ectopic positions (Figures 1C,D), similar to those observed in the fruit organ of *sun* mutant (Panteris et al., 2011; Komis et al., 2017; Ovečka et al., 2020).

In *Arabidopsis*, IQD5 stabilizes microtubules and regulates pavement cell morphogenesis, possibly by controlling the rates of cellulose deposition in anticlinal cell walls (Liang et al., 2018; Mitra et al., 2019). Similarly, IQD16, also termed Abnormal Shoot 6 (ABS6), mediates cortical microtubule organization, and pavement cell expansion (Bürstenbinder et al., 2017), which may involve physical interaction with the microtubule severing factor KTN1 (Li et al., 2020). Interestingly, aberrant expression of many IQD proteins changes pavement cell shape. IQDs may affect anisotropic cell expansion by recruiting CaMs and CaM-Likes (CMLs) and/or KLCR/CMUs, and rearrange the microtubule cytoskeleton topology and cellulose deposition not only during pavement cell development (Figure 1C) (Bürstenbinder et al., 2013; Mitra et al., 2019). Altered abundance of IQDs thus may override stress-derived growth patterns and manipulating *IQD* expression levels may provide a promising strategy in *de novo* domestication approaches to generate fruit organs with altered shape. Additionally, fruit shape is also partially regulated by proteins of the TRM (TONNEAU1 Recruiting Motif) family, which are subunits of a TTP (TON1-TRM-PP2A) complex (Spinner et al., 2013). Overexpression or loss-of-function of *TRMs* in rice and tomato changes cell elongation and cell division, producing elongated or shortened grains and fruits (Wang et al., 2015; Wu et al., 2018).

## Conclusion and Future Perspective

The regulation of microtubule organization is affected by multiple signals, such as plant hormones (e.g., ethylene, brassinosteroids), mechanical forces, and light (Ma et al., 2018; Ruan et al., 2018), which we have not discussed here in detail because of length constrains. It is noteworthy that plant hormones are commonly applied in agriculture. Thus, manipulating microtubule function precisely through exogenous application of these hormones may provide an alternative approach for crop cultivation, fruit shape and quality establishment. On the other hand, current studies on the function of microtubules in cell morphogenesis mainly rely on simple experimental systems, such as root and leaf pavement cells (a two-dimensional system). The real situation in fruits and other complex-shaped organs might be different. As each individual cell can sense the pressure and stereo-hinderance generated by neighboring cells, such feed-back mechanisms also affect cellular heterogeneity and microtubule organization (Long et al., 2020). Therefore, further studies of cytoskeleton structure and dynamics in more complex tissues, although challenging, will certainly advance our knowledge in the field.

## Author Contributions

ZB, ZX, and PW wrote the manuscript. ZB and JZ drew the schematic diagram. ZX arranged the references. KB and PW proposed some suggestions and modification opinions. All authors contributed to the article and approved the submitted version.

## Conflict of Interest

The authors declare that the research was conducted in the absence of any commercial or financial relationships that could be construed as a potential conflict of interest.
